# Phyto‐fabrication, purification, characterisation, optimisation, and biological competence of nano‐silver

**DOI:** 10.1049/nbt2.12007

**Published:** 2021-02-02

**Authors:** Bashir Ahmad, Farah Shireen, Abdur Rauf, Mohammad Ali Shariati, Shumaila Bashir, Seema Patel, Ajmal Khan, Maksim Rebezov, Muhammad Usman Khan, Mohammad S. Mubarak, Haiyuan Zhang

**Affiliations:** ^1^ Center of Biotechnology and Microbiology University of Peshawar Peshawar Khyber Pakhtunkhwa Pakistan; ^2^ Department of Chemistry University of Swabi, Swabi Anbar Khyber Pakhtunkhwa Pakistan; ^3^ K.G. Razumovsky Moscow State University of Technologies and Management (The First Cossack University) Moscow Russian Federation; ^4^ Department of Pharmacy University of Peshawar Peshawar Khyber Pakhtunkhwa Pakistan; ^5^ Bioinformatics and Medical Informatics Research Center San Diego State University San Diego California USA; ^6^ Oman Medicinal Plants and Marine Products University of Nizwa Nizwa Oman; ^7^ V.M. Gorbatov Federal Research Center for Food Systems of Russian Academy of Sciences Moscow Russian Federation; ^8^ A. M. Prokhorov General Physics Institute Russian Academy of Science Moscow Russian Federation; ^9^ Bioproducts Sciences and Engineering Laboratory (BSEL) Washington State University Richland Wasington USA; ^10^ Department of Energy Systems Engineering Faculty of Agricultural Engineering and Technology University of Agriculture Faisalabad Pakistan; ^11^ Department of Chemistry The University of Jordan Amman Jordan; ^12^ Changchun Institute of Applied Chemistry Chinese Academy of Sciences Changchun China

## Abstract

Published studies indicate that virtually any kind of botanical material can be exploited to make biocompatible, safe, and cost‐effective silver nanoparticles. This hypothesis is supported by the fact that plants possess active bio‐ingredients that function as powerful reducing and coating agents for Ag+. In this respect, a phytomediation method provides favourable monodisperse, crystalline, and spherical particles that can be easily purified by ultra‐centrifugation. However, the characteristics of the particles depend on the reaction conditions. Optimal reaction conditions observed in different experiments were 70–95 °C and pH 5.5–8.0. Green silver nanoparticles (AgNPs) have remarkable physical, chemical, optical, and biological properties. Research findings revealed the versatility of silver particles, ranging from exploitation in topical antimicrobial ointments to in vivo prosthetic/organ implants. Advances in research on biogenic silver nanoparticles have led to the development of sophisticated optical and electronic materials with improved efficiency in a compact configuration. So far, eco‐toxicity of these nanoparticles is a big challenge, and no reliable method to improve the toxicity has been reported. Therefore, there is a need for reliable models to evaluate the effect of these nanoparticles on living organisms.

## 1 INTRODUCTION

Nanotechnology is the study of extremely small entities, and it is interdisciplinary in nature including material science, biological, biotechnology, and engineering [1]. The value of metal NPs was USD 10.92 Billion, in 2016, and it is projected to increase up to USD 25.26 Billion, in 2022 [2]. Among the array of NPs, metallic AgNPs have significance in multiple areas such as in bio‐labelling, biosensors, and filters [3,4]. Their antimicrobial potency makes these particles efficient bio‐remediators, water purifiers, and antibiotics [5, 6, 7]. In this respect, several physical and chemical methods are available for the preparation of nano‐silver. However, these methods are prone to various snags such as elevated cost and energy dissipation, in addition to the use of certain noxious reagents as passivators (a type of corrosion inhibitors), particularly mercapto‐acetate, thiourea, and thiophenol [1,8,9].

Physical methods are more attractive because they lead to high purity AgNPs as compared with products of other methods. However, the primary hindrance in the application of these methods at an industrial scale is their high‐energy requirements and lower yield. Consequently, the green synthesis of metallic NPs is gaining attention among scientists because these nanoparticles are cheaper, easy to synthesis, and are eco‐friendly [10]. Moreover, the green synthesis of NPs can be easily scaled up to produce large quantities. In addition, no toxic stabilizers and reductants are involved during the green synthesis of AgNPs [11]. Furthermore, the principals of green chemistry are used for the fabrication and designing of the nanoscale products to avoid their harmful effects due to the extensive use of these products [12]. For the green synthesis of these nanoparticles, polysaccharides, bacteria, fungi, and plant extracts are used as stabilisers and reducing agents [13]. In this context, plant extracts are a very simple method for making nanoparticles because it does not involve complex process of preserving and culturing biotic cells. Moreover, silver nanoparticles obtained using plant extracts are harmless for the therapeutic practices in humans [14,15]. In addition, stabilisation, fabrication, and reduction of AgNPs using plant extracts can be achieved by using the biochemical constituents present in these extracts. Therefore, plants are considered as one of the best resources for large‐scale synthesis of metal oxides and metal nanoparticles with distinct size and morphology [16].

Recent findings revealed developed and exciting biological procedures to generate nano‐silver via microbes or botanic materials as potential bio‐reducers and bio‐cappers [13,17]. Along this line, AgNPs are used for precise bimolecular detection, antimicrobials, and diagnostics [18]. These particles possess several unique properties, which make them gaining the attention as antifungal, larviciadal, antibacterial, and antioxidant agents [19,20]. They are also being used in the field of solar cells, agriculture, pollution control, waste management, medicine, and forensic science [21]. In comparison with microbial biosynthesis, phyto‐synthesis has furnished a novel dimension to material chemistry by manufacturing biocompatible silver nano‐structures. This method is green, economical, and reproducible, due to the diversified availability of the materials, ease of manipulation, and minimal impediments [22, 23, 24, 25, 26, 27, 28]. In this regard, phyto‐synthesised AgNPs containing alkaloids and flavonoids can be used for bactericidal activity of pathogens found in humans [15]. Microbes such as bacteria and fungi have dynamic capacity to reduce silver ions (Ag^+^) to particles (Ag^o^), however, more investigations are required to reap crystalline, monodispersed, and uniform conformation. In addition, isolation and purification of intra‐ and extra‐cellular synthesised microbial nano‐silver from the biological system is an immense challenge [29,30]. Based on the above discussion, herein, it is designed to assess the phyto‐synthesis of nano‐silver, their multiple approaches of purification, characterisation, and optimisation, and finally their applications and toxicity in medical, industrial, and environmental sectors.

## 2 PHYTO‐FABRICATION OF NANO‐SILVER

**FIGURE 1:**
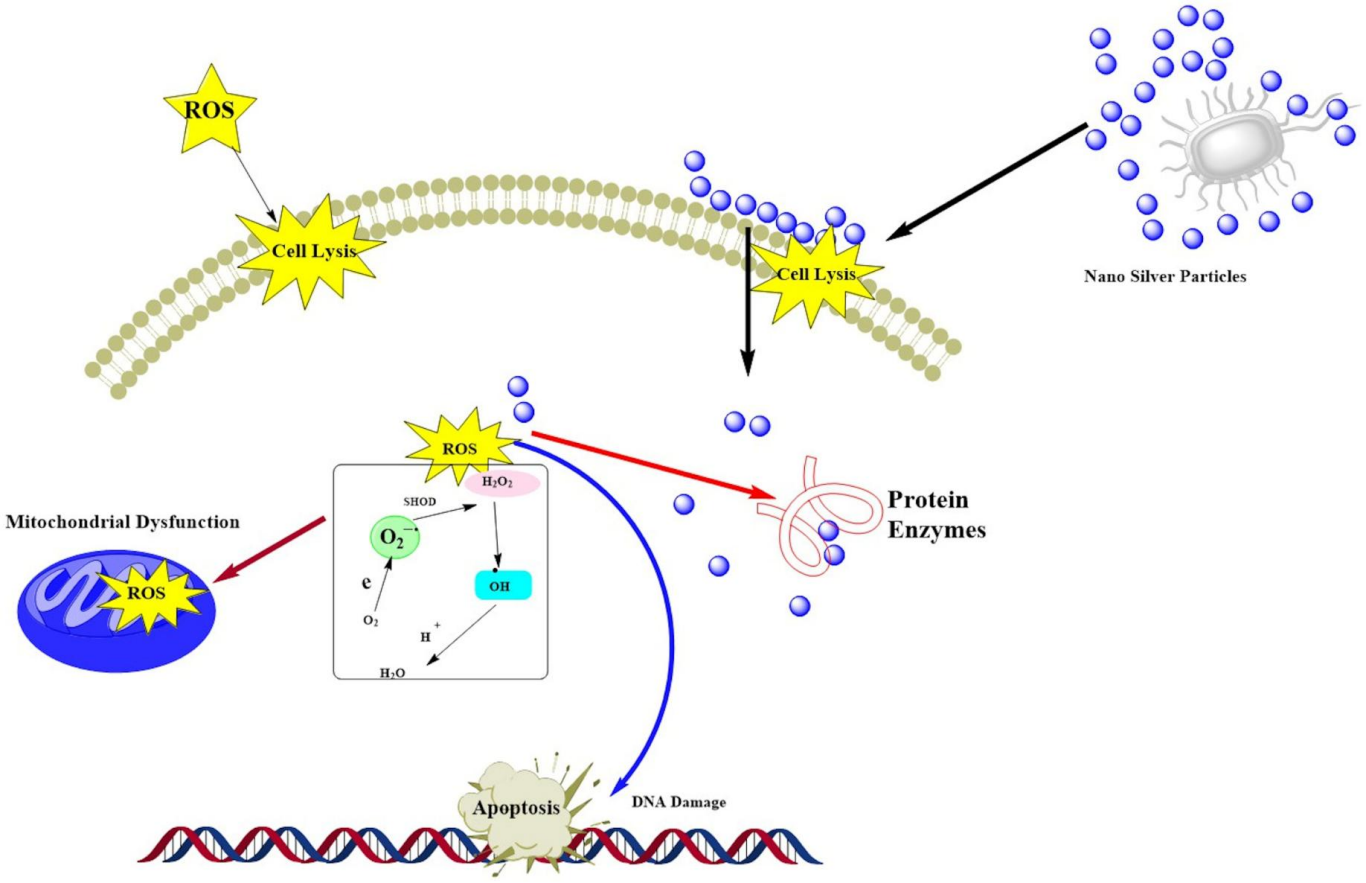
Schematic of antimicrobial activity associated with Ag nano‐silver

According to the literature, phyto‐compounds that act as reducing agents for conversion of Ag + to Ag^o^ are polysaccharides, proteins, polyphenols, alkaloids, and flavonoids, among others. These biomolecules occur in significant amounts in plant parts such as leaves, roots, stem, bark, flowers, and fruits. Biocompatible AgNPs were earlier mass produced using leaves broth of *Acalypha indica* (Indian nettle) [31], *Camellia sinensis* (tea) [32], *Capsicum annuum* (chili) [33], *Cymbopogon flexuosus* (lemon grass) [34], *Datura metel* (devils trumpet) [34], *Medicago sativa* (alfalfa) [34], *Pelargonium graveolens* (geranium) [34], *Diospyros kaki* (persimmon) [35], *Ginkgo biloba* (maiden‐hair tree) [36], *Magnolia kobus* (Kobushi) [35], *Pinus densiflora* (pine) [15], *Platanus orientalis* (oriental plant) [35], *Euphorbia hirta* (asthma‐plant) [36], *Eucalyptus citriodora* (lemon eucalyptus) [37], *Ficus benghalensis* (Figure 1) [37], *Garcinia mangostana* (mangosteen) [38], *Ocimum sanctum* (basil) [38], *Acacia nilotica* [39], *Cyperus conglomeratus* [40], *Ziziphus mauritiana* Lam. [41], *Sesbania grandiflora* [42], *Psidium guajava* [43], aqueous root extracts of *Arachis hypogaea* (ground nut) [44], *Catharanthus roseus* (Madagascar periwinkle) [45], *Mammea suriga* (surangi) [46], *Cannabis sativa* (marijuana) [47], *Vetiveria zizanioides* (vertiver) [47], and *Zingiber officinale* (andraka) [48]. Broth from the bark of *Cacumen platycladi* (Thujae), *Cinnamon zeylanicum* (cinnamon), *Cochlospermum gossypium* (gum kondagogu), and *Pinus eldarica* (Elder) also acts as potential ionic silver reducers [49].

In a similar fashion, aqueous flower extracts from *Couroupita guianensis* [50], *Nyctanthes arbortristis* [51], and *Tagetes erecta* (Marigold) [52], *Cananga odorata* [53] have been verified to phyto‐synthesise biocompatible nano‐silver particles possessing good antimicrobial properties. Furthermore, aqueous fruit extracts from *Andean blackberry* [54], *Citrus limon* [55], *Citrus reticulate* [55], *Citrus sinensis* [55], *Tanacetum vulgare* (tansy fruit) [56] and *Vitis vinifera* (grape vine) [57], *Artemisia nilagirica* [58], *Phyllanthus reticulatus*, and *Conyza bonariensis* [59], *Ficus retusa* [60], *Momordica charantia* [61], *Ocimum gratissimum* [62], *Ficus benghalensis* [63], *Elephantopus scaber*, and *Azadirachta indica* [64], and *Gardenia jasminoides* Ellis [65] were reported to contain compounds that can reduce silver ions. On the other hand, Eucalyptus leaf extract [66], *Coccinia grandis* fruit extract [67], Reetha and Shikakai leaf extract [68], *Alchornea laxiflora* leaf extract [69] were also documented to have reducing phyto‐compounds that aid in the fabrication of silver nanoparticles which can be exploited for various purposes. Table 1 summarizes the studies on phytofabrication potentials of various medicinal herbs.

**TABLE 1:** Summary of the literature on phytofabrication studies, the applied plants along with the targeted applications

Plants	Plant Part	Size (nm)	Shape	Application	Reference
*Annona muricata*	Fruit	60.12	Spherical	Antimicrobial	[70]
*Portulaca oleracea* L	Stems, leaves and roots	175,136,146	Multiple	N.D	[71]
*Allium giganteum*	Shoots	12	Spherical	Antibacterial and anticancer	[72]
*Allium saralicum*	Leaves	‐	‐	Antioxidant, antibacterial and antifungal	[73]
*Tropaeolum majus* L.	Leaves	35‐55	Round	Antioxidant, antibacterial, antifungal and anticancer	[74]
*Piper betle*	Leaves	3‐37	Spherical	Medical and pharmaceutical	[75]
*Allium sativum*	Root	7.3	Spherical	Antibacterial	[76]
*N. jatamansi*	Rhizomes	10‐15	Round	Anti‐inflammatory and ant biofilm	[77]
*Annona reticulata*	Leaves	6‐8	‐	Antibacterial	[78]
*Justicia spicigera*		80‐‐100	Spherical	Antimicrobial	[79]
*Salvia hispanica* L	Seeds	1‐30	Spherical	Antibacterial	[80]
*Pinus densiflora*	Leaves	15‐50	Cubic	Cosmetics, foods, and medical	[35]
*Pithecellobium dulce*	Leaves	62	Spherical rods	Larvicidal	[81]
*Falcaria vulgaris*	Leaves	A rane	‐	Antibacterial	[82]
Tarragon	Leaves	25	Spherical	Antibacterial	[83]
Selaginella	Leaves	5‐10	Round	Anti, icrobial	[84]
*Rumex dantatus*	Root	25‐70	‐	Antibacterial	[85]
*Coptis chinesis*	Leaves	6‐45	Round	Chemotherapy	[86]
*Aloe vera*	Gel	66.6	Spherical	Antioxidant	[87]
Anthoceros	Whole plant	20‐50	Cuboidal and triangula	‐	[88]
*Salvia officinalis*	Leaf	41	Spherical	Antiplasmodial	[89]
*Morus nigra*	Leaves	23	Cubical	Antibacterial	[90]
*Helianthus annus*	Leaves	19	Multiple	Antibacterial	[91]
*Memecylon umbellatum* Burm F	Leaves	7‐23	Round	Antibacterial/antitumour	[92]
*Calotropis gigantean*	Leaves	83.7, 5.9,11.8	Spherical	Antibacterial	[93]

The synthesis of NPs using a single active substance is getting attention of scientists because it has been observed that the purification of such particles is easy. However, further research is required for the applications of such particles in medical fields. Some recent studies have shown that flavonoids present in plant extracts are the main compounds, which contribute the bio reduction of metal ions to nanoparticles. The phytochemicals such as alkaloids, proteins, phenols, alcohols, terpenoids, and flavonoids are generally present in plant materials, and it has been observed that these materials are actively involved in stabilisation and reduction of metal ions [94,95].

### 2.1 Mechanism of Photosynthesis of AgNPs

Numerous studies suggested the synthesis of NPs [96, 97, 98, 99], however, the exact mechanism about synthesis is still not known. Several studies were carried out at the National Chemical Laboratoy (NCL), Pune, India, to find the mechanism for the synthesis of silver and gold nanoparticles from different plants including chickpea [100], neem [101], tamarind [102], and lemon grass [103]. They reported that terpiniods present in plant material are the main compounds that contribute to reduction of silver ions. Moreover, they also reported that proteins are the main capping agents in gold nanoparticles [104]. When the *Azadirachta indica* leaf extract was used for synthesising metallic and bimetallic nanoparticles, it was observed that reducing sugars are the main components involved in reduction of metal ions as well as stabilisation of these nanoparticles [101]. Similarly, reducing susgars were also involved in the formation of AuNPs from lemon grass [103].

### 2.2 Factors affecting the synthesis of nanoparticle

There are many factors that influence the synthesis of NPs including temperature, pressure, light intensity, and incubation time. Moreover, some other factors including concentration of leaf extract, concentration of AgNO_3_, reaction time, reaction temperature, pH of the reaction, and other light reactions affect the size, dimension, and shape of these NPs. In addition, the conditions of light such as tube light, blue light, bulb light, and red light also influence the synthesis of these NPs.

### 2.3 Temperature

Temperature is the main factor, which affects the synthesis of AgNPs. Generally, synthesis of nanoparticles takes place at room temperature, which is a time‐consuming process. However, the temperature can be increased to lower the time. In general, the synthesis is performed at a temperature ranging from 30 to 100°C. The synthesis at higher temperature results in reduction of Ag^+^ ions as well as homogeneous nucleation of silver nuclei, resulting in smaller size silver NPs [105]. The rate of NPs synthesis decreases and the stability increases with the increase of reaction temperature. Moreover, the size of the silver NPs decreases with the increase of reaction temperature.

### 2.4 Volume of extract

The volume of leaf extract affects the time required for the formation of NPs as the leaf extract is the main part of reaction of silver ion. Usually 1, 5, 10, 15, 20, and 100 ml of extract volume can be used to analyse the effect. The volume of leaf extract affects the particle size of the silver NPs. The reduction of Ag^+^ ions is significantly affected by the concentration of leaf extract that is the contents of anthocyanin, phenol, tannins, polyphenols, and polysaccharides [106].

### 2.5 pH

pH can affect the electrical loads of biomolecules which consequently can affect their stability, capping ability as well as their growth. Research findings indicated that the acidic pH is not suitable for the synthesis of NPs because under acidic conditions the transition from light green to dark brown takes longer time. The size of the NPs formed under acidic conditions is usually larger as compared with‐ the ones synthesised under basic conditions. Moreover, the alkaline conditions also favour reduction as well as the stabilising capacity of antioxidants [106].

### 2.6 Silver nitrate (AgNO_3_) concentration

The AgNO_3_ concentration is measured in 0.1–1 mM or higher concentrations such as 10, 50, and 100 mM. However, 1 mM concentration has been found best as it was observed previously that the particles synthesised using 0.1 mM AgNO_3_ are very minute and cannot be seen with a naked eye. Therefore, reactants are required in very small concentrations. The higher concentrations of reactants results in an unsuccessful reduction of Ag^+^.

### 2.7 Stirring time of reaction

It is the time required for the synthesis of silver NPs, starting from the reactants added to beaker. The stirring time allow the proper reaction between reducing complex components of leaf extract with silver salt. If the concentration of phytochemicals or secondary metabolite is higher in a plant, it will reduce the silver salt in less time and vice versa. The NPs formation takes place quickly if the plant contains less secondary metabolites [106].

## 3 PURIFICATION OF NANO‐SILVER

There are numerous purification techniques, which are currently used for the purification of NPs. These techniques include centrifugation, precipitation by antisolvent, flocculation using photolyzable surfactant, or temperature‐induced separation. However, most of these techniques are time consuming [107]. Moreover, they require higher amounts of solvent as well as higher energy, and they are inefficient in removing small molecule and inorganic impurities [108,109].

Published research indicated that the recommended procedure for isolation and purification of nano‐silver from a complex reaction mixture is ultra‐centrifugation at high speeds (200,000 rpm) for 15 min, or sucrose density gradient centrifugation [109,110]. In addition, a new technique termed as differential thawing was recently introduced for the purification of nano‐silver, which involves the freeze‐drying of prepared nano‐silver solution in a deep freezer overnight, and then thawing the icy nano‐silver at 28 °C. However, centrifugation is a more preferable approach than differential thawing because the latter yields larger and polydispersed particles due to agglomeration [111].

Multifarious purification protocols that provide tremendous outcomes depending on the feasibility of the wet laboratory have been investigated. Out of many, sucrose density gradient centrifugation, flow centrifugation, and repeated ultra‐centrifugation at 10,000 rpm for 10 min are preferred, due to their ease in handling, less laborious, speedy, and budget‐friendly results. According to earlier studies, green nanoparticles from leaves of *Eugenia jambolana*, *Piliostigma thonningii*, and *Azadirachta indica* were purified following sucrose density centrifugation method which provided purified AgNPs in estimated range of 10–100 nm [112,113]. In an another study, crude phyto‐AgNPs from *Tagetes erecta* flowers were purified following ultracentrifugation for 10 min at ≤ 50F0B0C. The repeated centrifugation for 10 min yielded excellent pelleted particles, which were dried and stored at 4F0B0C. The purified pellets were characterised to be monodispersed, polycrystalline, and almost spherical in morphology owing to a size of about 1–90 nm, thus qualifying them as ultra‐fine particles [52]. Purification of nanoparticles is a crucial step for proper characterisation and function of green AgNPs. The excess ligand and impurities hinder the precise function of AgNPs. To cope all this, a novel strategy of continuous flow purification was introduced in February 2020, which claimed to eliminate 56.73% of the hindering ligands while maintaining the desired structure of biosynthesised AgNPs. In addition, the purified AgNPs can be effortlessly dried via freeze‐drying (–20F0B0C or below) or spread drying (≤50°C) method [114].

## 4 CHARACTERISATION OF NANO‐SILVER

Nano‐silver is characterised by numerous spectroscopic techniques including UV–Vis spectroscopy, scanning electron microscopy (SEM), energy dispersed X‐ray spectroscopy (EDX), X‐ray diffraction spectroscopy (XRD), transmission electron microscopy (TEM), atomic force microscopy (AFM), zeta potential, dynamic light scattering (DLS), DLS, energy‐dispersive spectra (EDS), nuclear magnetic resonance spectroscopy (NMR), UV–Vis spectroscopy, surface‐enhanced Raman spectroscopy, high‐resolution transmission electron microscopy (HR‐TEM), AFM, X‐ray photoelectron spectroscopy (XPS), Fourier transform infrared spectroscopy (FTIR), and surface‐enhanced Raman spectroscopy [115,116]. These techniques are useful in investigating the precise and controlling synthesis of AgNPs. Parameters such as surface area, morphology, topology, porosity, crystallinity, dispersion, and the size of a sole particle are analysed via these techniques. In addition, nanocomposites, nanotubes, and nanowires orientation and intercalation can be determined by means of the aforementioned techniques [117]. For instance, the diameter and conformation of nano‐silver are investigated by SEM, TEM, and AFM, which normally varies from 10 to 500 nm in size having complex morphology of spherical to complex rods, triangles, trapezoids, and prisms. On the other hand, the crystalline structures are probed by XRD, whereas size distribution is assessed by DLS.

Furthermore, EDX spectroscopy can be used to investigate the elemental status of biogenic nano‐silver, whereas UV–Vis spectrophotometry confirms the explicit sample formation by scrutinizing absorbance values based on *λ*_max_ showing plasmon resonance in the range of 350–500 nm [118]. In this respect, Saratale et al. used *Acacia nilotica* leaf (ANL) extract to produce silver nanoparticles. In this case, the involvement of phytoconstituents was analysed using energy‐dispersive spectra (EDS) and Fourier transform infrared spectroscopy (FTIR) which confirms the involvement of ANL in stabilisation, reduction, and capping of the silver nanoparticles matrix. Characterisation of silver nanoparticles was carried out with the aid of X‐ray diffraction (XRD) and high‐resolution transmission electron microscopy (HR‐TEM); results showed that the average particle size of the silver nanoparticles was ∼20 nm with spherical shape and crystalline structure [39].

## 5 OPTIMISATION OF NANO‐SILVER PARTICLES

Optimising crucial parameters such as substrate concentrations, pH, and temperature are the principle keys to obtain accurate, monodispersed, crystalline, and nano‐sized particles. A study by Ahmad and colleagues showed that the substrate concentration is directly proportional to the rate of reaction [119]. An increase in substrate concentration from 1 to 5 mM AgNO_3_, caused a rapid nano‐silver synthesis, however, the stability was more controlled at 1 mM substrate concentration; where stable, monodispersed, and small particles of AgNPs were produced at an average size of 10 nm. In comparison, higher substrate concentrations led to the formation of unstable, polydispersed, large aggregated nano‐silver with a diameter of ≥100 nm [63]. Similarly, the pH and temperature of reaction mixture are also considered as prime factors for precise phytosynthesis of nano‐silver. It was shown that green AgNPs obtained at temperatures ≥100°C and pH 4, were unstable large particles of size ≥55 nm. The favourable conditions that assist in brisk biosynthesis were temperature 70–95°C and slightly acidic to neutral pH that is 5.5–8, which produces small nanoparticles with increased surface area [63,120].

### 5.1 Biocompatibility of AgNPs

Nano‐silver has garnered prodigious attention across the globe because of their dynamic applications in multifarious spheres. Their unique physicochemical characteristics have been applied in several biomedical devices ranging from a simple wound dressing to complicated pacemakers. Their multiple modes of action along with cost effective, eco‐friendly and least toxic nature had made their utilisation unlimited because of their promising outcomes [121]. Along with many pros of green AgNPs, there are certain controversies arising in regard to their biocompatibility with normal animal cells peculiarly to humans. Various present day studies suggest that sustained exposure to AgNPs can cause their accumulation in vital organs and complicate the therapeutic scenario [122, 123, 124]. It is evidenced that inhaled AgNPs may cause lung injuries by damaging the alveoli. Similarly, deposition of AgNPs is also reported in nervous, cardiac, renal, and hepatic tissues [125, 126, 127]. Cytotoxic evaluation evinced that AgNPs have potential to bio‐transform the natural environment of living cells by generation of oxidative stress that interact with biological macromolecules [128,129]. In another molecular analysis, it was manifested that AgNPs have the capability to modify mitochondrial membrane permeability and accumulation of reactive oxidative species which in turn cause profuse DNA damage [130,131]. Many studies proved that AgNP exposure can induce a decrease in cell viability through different cellular mechanisms. One of these mechanisms is represented by the induction of apoptosis‐related genes and the activation of apoptosis mechanism. Also, it was proven that nano‐silver can cause the formation and intracellular accumulation of ROS, modification of mitochondrial membrane permeability, and DNA damage [132]. The in vitro toxicity of AgNPs has been investigated in several research studies, but there is still a lack of consistent and reliable data. Furthermore, genotoxic, cytotoxic, and mutagenic investigations validated that toxicity of biogenic AgNPs is related to surface chemistry, morphology, size, therapeutic concentration, dispersion rate, and physicochemical characteristics [133]. In vivo toxicity of AgNPs is categorised according to the site of impairment that is caused. The various modes of exposure to AgNPs, their biodistribution, and their mechanisms underlying the effects are documented in literature [133]. As AgNPs are preferably utilised in most of the modern day technology, the continuous exposure may pose an alarming condition to human health. There is a consistent requirement of more cytotoxic and genotoxic research to be conducted to understand the underlying side effects of the phyto‐fabricated silver nanoparticle [134, 135, 136].

### 5.2 Stability of AgNPs

Stability of AgNPs can be improved by applying different analytical techniques. These techniques include fluorescence correlation spectroscopy (FCS), DLS, flow field fractionation (FFFF), AFM, and nanoparticle tracking analysis (NTA). The area stabilisation and surface charge of AgNPs can be understood using Zeta potential and BET surface area measurements. Besides surface charge, steric stabilisation of AgNPs could also occur [137]. The stability of AgNPs in aqueous solutions is affected by the coating of chemical materials. Most of the stability analyses of AgNPs have been done using citrate‐coated AgNPs [138, 139, 140, 141]. A previous study on the stability of citrate‐coated AgNPs in synthetic seawater, natural freshwater, and stimulated estuarine waters has shown a stability of few days of these materials [138].

### 5.3 Stability of Phytofabricated AgNPs

Phyto‐synthesised AgNPs were investigated for their long‐term stability at variable fluctuation conditions. Aqueous extracts from *Parachlorella kessleri* were utilised to synthesize AgNPs. Different spectrophotometric analyses demonstrated that the rate of synthesis is directly proportional to freshness of sample and amount of AgNPs solution. From scanning and transmission electron microscopy, it was concluded that a pH range of 8–10 provided fine, stable monodispersed polyhedron particles in the diameter of 5–60 nm. In contrast, acidic pH of reaction afforded minimally stable particles of irregular shape and size, thus losing their integrity at 10th day of the experiment. In addition, temperature and light fluctuations also play a vital role in the stability of greenly synthesised nano‐silver. Reactions temperature below 20F0B0C and excessive interaction of light tend to disintegrate the stability of potential nanoparticles. Furthermore, storage temperature of ≤5°C provides best long‐term stability. The AgNPs remained desirably spherical, ultra‐fine (5–20 nm), and stable without agglomeration even after more than 6 months [142,143].

### 5.4 Reproducibility of Phytofabricated AgNPs

Biofabrication of silver nanoparticles by biological reducing agents including plant, bacterial, and fungal extracts is a substitute to chemical and physical methods, although preparation of bacterial and fungal extracts requires high maintenance of cultures and are time consuming as compared with plants. Many plant species, algae and their endophytes such as *Averrhoa carambola*, *Solidago altissima*, *Chlorella vulgaris*, *Calophyllum apetalum*, and *Padina tetrastromatica* have been successfully used for the synthesis of AgNPs [144]. Phytofabrication of AgNPs using aqueous extracts from whole parts or plant parts showed promising medicinal potency in remediating many ailments such as malaria rheumatism, microbial infections, leprosy, and even cancer. Plant extracts provide bioactive ingredients, which act as stable bio‐factories for recyclable and reproducible synthesis of AgNPs in an economic and eco‐friendly manner with aced medicinal properties [145].

### 5.5 Dispersity of Phytofabricated AgNPs

Biogenic AgNPs are considered to be amphipillic particles because of their affinity towards both polar and non‐polar solvents. According to various biological investigations, dissolution of AgNPs in number of solvents ranging from water to *n*‐hexane has broadened up multiple experimental vistas to work on and to make the phyto‐fabricated AgNPs commercially available for multitude of products [145].

### 5.6 Uniform size of Phytofabricated AgNPs

According to a present day investigation, it is validated that fine control over the nanoparticle's size is achieved by varying the concentration of tannic acid (a phytoactive compound), which results in uniform nanoparticles in the range of 18–30 nm in diameter with a standard deviation of less than 15%. Phyto‐reduction method is a preferred route because of profuse amounts of eco‐friendly and cost effective bio‐reducing and bio‐capping agents, which provide the desired morphology. It is also known that utilisation of various stabilizing agents such as sodium borohydride, polyvinylpyrrolidone, and tri‐sodium citrate provides uniform spherical morphologies in the diameter of about 15–90 nm. It was also observed that spherical AgNPs exhibited a better antibacterial activity because of their proficient mode of penetration plus multiple target affinity [146, 147, 148, 149, 150].

## 6 BIOLOGICAL COMPETENCE OF NANO‐SILVER

In the past few years, explorations on green AgNPs have expanded to diverse fields such as medicine, agriculture, environment, and many other industries [2]. Documented applications of phyto‐based AgNPs in the above‐mentioned domains are discussed in the following sections.

## 7 MEDICINAL APPLICATIONS

### 7.1 Antimicrobial

Centuries ago, silver was acknowledged as a notable antimicrobial agent whether in the form of colloids, salts, or pure solutions. This attribute was inherited in AgNPs with superior characteristics. Among all of the metals which are considered as anti‐bacterial agents, AgNPs have been proven to be the most effective for a wide range of microorganisms [151, 152, 153]. These particles have the ability to control the microbial growth in human infection. The antimicrobial effectiveness of silver nanoparticles mainly depends on their size and shape. The smaller size AgNPs are more effective as an antimicrobial agent as compared with the larger one. According to previous reviews, AgNPs is a broad‐spectrum antibiotic that inhibit pathogenic microorganisms. The amount of green AgNPs is directly proportional to the antimicrobial effect. If the size of AgNPs is smaller, the antimicrobial prospects will be superior due to higher affinity and extended surface area towards microbial membranes [154]. Conformation also plays a crucial part in contributing to antimicrobial property. Criterion was tested by Sadeghi and coworkers who postulated that nanoplates are excellent bactericidal agents against *Escherichia coli* and *Staphylococcus aureus* [155]. Previous experiments hypothesised that a combination therapy of AgNPs with antibiotics such as amoxicillin can be utilised to manage resistant microbial infections [156]. Similarly, findings by Kim and coworkers revealed that the combination therapy restrains the mycelia growth of several resistant fungi such as Candida and Trichophyton species [157].

AgNPs have the potential to kill both gram‐positive and ‐negative bacterial strains [158, 159, 160]. However, nanoparticles are considered more effective for killing gram‐negative bacteria as compared with the gram‐positive bacterial strains. This could be attributed to the fact that the gram‐positive bacteria have a thick cell wall consisting of different peptidoglycan layers 920–80 nm) in addition to one cytoplasmic membrane. On the other hand, gram‐negative bacteria contain an external layer of lipopolysaccharide (LPS) followed by peptidoglycan, which is a very thin layer, and the last layer is of plasma membrane [161,162]. Multi‐drug bacteria, which are now well recognised, have become a major threat to the human health [163]. The coating of AgNPs with hydrophobic and cationic functional groups is helpful in suppressing the growth of gram positive, gram negative, and multi‐drug‐resistant bacteria [164]. AgNPs are also effective in overcoming these multi‐drug bacteria. The AgNPs release Ag^+^ ions during their antibacterial activity, which interact with bacterial protein particularly the thiol group. Due to this interaction, the DNA replication gets disturbed which results in lysis of bacteria [165, 166]. The antimicrobial activity of the AgNPs depends upon the size, dose, charge, and shape [167,168].

Antimicrobial mechanisms of biogenic AgNPs is not conventionally elucidated, but it is inferred that nano‐silver possesses affinity towards almost all microorganisms. These particles have the capacity to induce cell membrane permeability via (a) free radical emergence or structural alterations, (b) inhibition of DNA replication via the inhibition of vital enzymes or phosphate containing bases, and (c) modulation of signal transduction to alter phosphotyrosine profile of microbial peptides. All these changes incite microbial cell apoptosis, thus conferring bactericidal and fungicidal effects (Figure 1) [169].

Silver nanoparticles are capable of binding with the cell wall of bacteria and then penetrate inside the cell. After penetration, they cause changes in cell wall structure, which includes permeability as well as death of the cell [144]. These particles enter by endocytosis after accumulating on cell surface [170]. Particles also have the ability to form free radicals, which cause death of the cell. Some previous studies have shown the formation of free radicals by silver nanoparticles through spin resonance spectroscopy during their contact with bacteria. These radicals then damage the cell wall and make them porous [171,172]. This causes the death of these cells. Soft bases have the capacity to react with soft acids. Cells are formed from phosphorous and sulphur, which are soft bases and the silver is soft acid. Sulphur and phosphorous are the major components of DNA and silver nanoparticles act on DNA and cause its destruction which ultimately cause the cell death [173].

Reactive oxygen species are also formed when silver particles encounter bacterial cell wall, which may be generated by the inhibition of respiratory enzymes by silver ions. They attack the cell and cause its death [174,175].

### 7.2 Antiviral

AgNPs have antiviral attributes and they are well known for their anticancer activity [176]. In this respect, infections of herpes simplex virus type 1 (HSV‐1) can be inhibited by AgNPs because these nanoparticles block the attachment and control the entrance of the virus into the cells. Moreover, they control the virus by preventing its spread from cell to cell. In addition, phyto‐generated AgNPs act as a robust antiviral agent against a wide array of strains such as human immunodeficiency virus type 1 (HIV‐1), HSV‐1, Hepatitis B virus (HBV), respiratory syncytial virus (RSV), and monkey‐pox virus [177,178]. Taylor and coworkers demonstrated that the antiviral efficacy of AgNPs is more intense than that of silver salt solutions. Silver salt solution releases only Ag^+^ ions, whereas the eco‐friendly nanoparticles solution imparts dynamic Ago (atomic) and Ag^+^ (ionic) clusters [179].

In a similar fashion, Lara and colleagues showed that AgNPs confine the replication of HIV‐1 at stage 1 by binding to viral glycoproteins, which halt the viral attachment, penetration, and fusion to CD‐4 T‐lymphocytes, hence inhibiting infection [180]. Shown in Figure 2a is the antiviral mechanism of AgNPs impact on HIV‐1 glycoprotein, whereas Figure 2b presents a table of amino acid residue related to HIV glycoprotein.

**FIGURE 2a:**
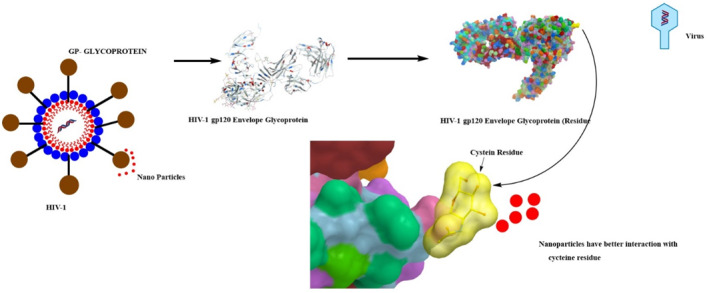
Antiviral mechanism of Ag naoparticles impact on HIV‐1 glycoprotein. Ag has the strongest interaction with cys residue

**FIGURE 2b:**
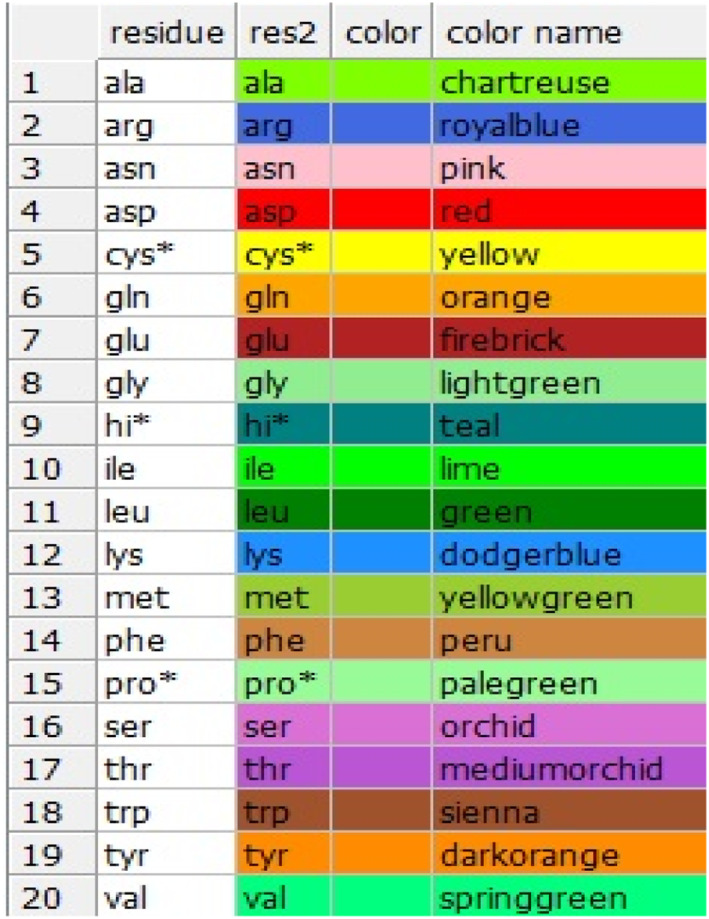
Colourful table of amino acid residue related to HIV glycoprotein, yellow colour is linked to cycteine residue

A recent study has also shown the potential of these NPs as a candidate for 2019‐nCoV treatment [181]. Zacher [182] proposed that the silver nanoparticles can be used to treat COVID‐19 at an early stage. The nanoparticles‐based vaccine is more effective in boosting immunity as compared with other conventional antigen‐based vaccines [181].

### 7.3 Anti‐inflammatory

**FIGURE 3:**
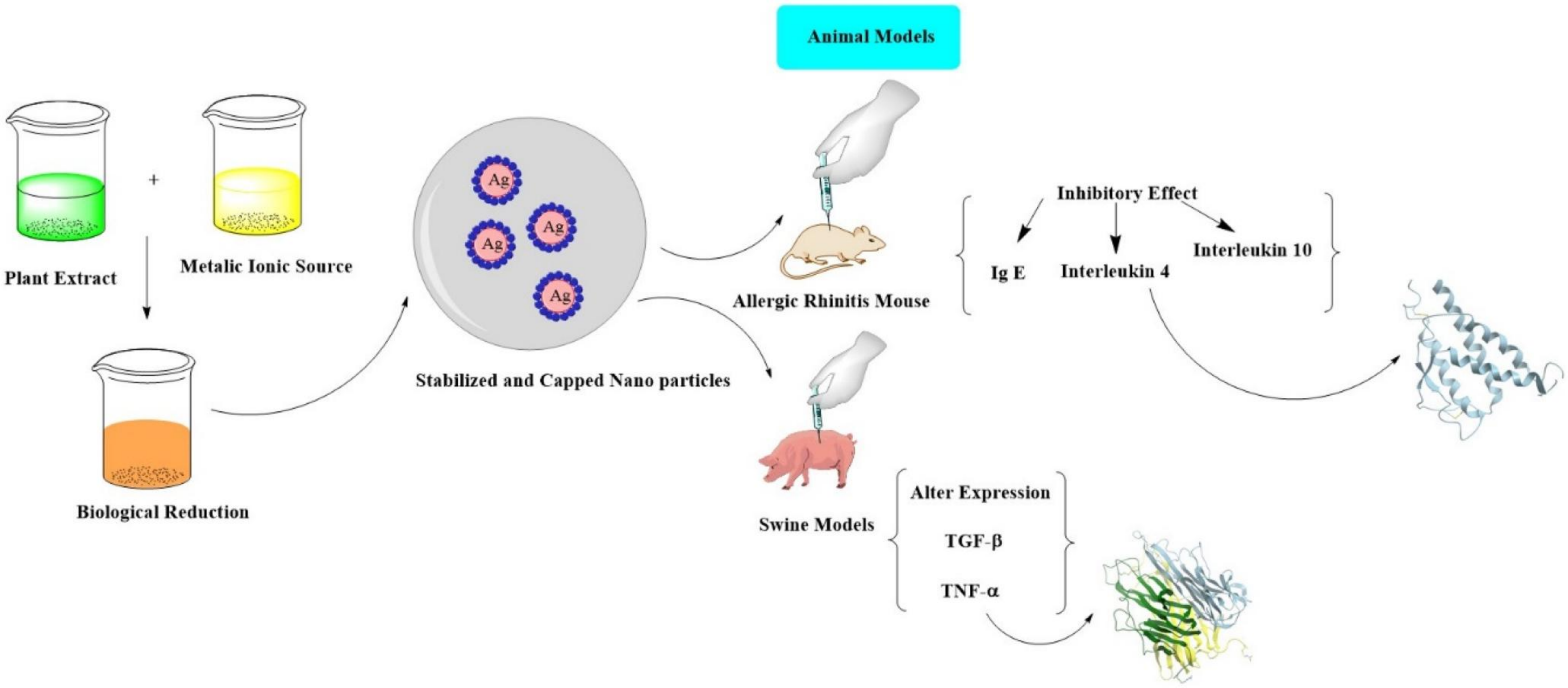
Production of Ag nanoparticle and its effect in two animal models; allergic rhinitis Mouse and Swine

Biosynthesised AgNPs were approved as an eminent anti‐inflammatory agent. Experiments involving animal models such as allergic rhinitis mouse revealed that the administered dose of botanic AgNPs inhibits the expression of ovalbumin‐specific immunoglobulin E, interleukin‐4 and ‐10, goblet cell hyperplasia, and inflammatory cell infiltration [183]. Contact dermatitis experimentations on swine models showed that administered doses of silver nanoparticles altered the expression of pro‐inflammatory cytokines tumour necrosis factor‐α (TNF‐α) and transforming growth factor‐β (TGF‐β) (Figure 3) [184]. Furthermore, clinical studies on humans showed anti‐inflammatory activity in response to chronic leg ulcers. The ameliorative effect of AgNPs occurred via the inflammatory cell apoptosis, reduction in the mast cells and lymphocytes infiltration, and by limiting the release of metalloproteinases and cytokines [185,186]. The anti‐inflammatory properties of the AgNPs are directly related to the size of these particles and larger diameter particles possess better anti‐inflammatory properties compared with the smaller one [187]. Moreover, the NPs prepared by the combination of extracts from different plants showed better anti‐inflammatory properties compared with the ones which were prepared by only one plant [188].

### 7.4 Wound Dressers

**FIGURE 4:**
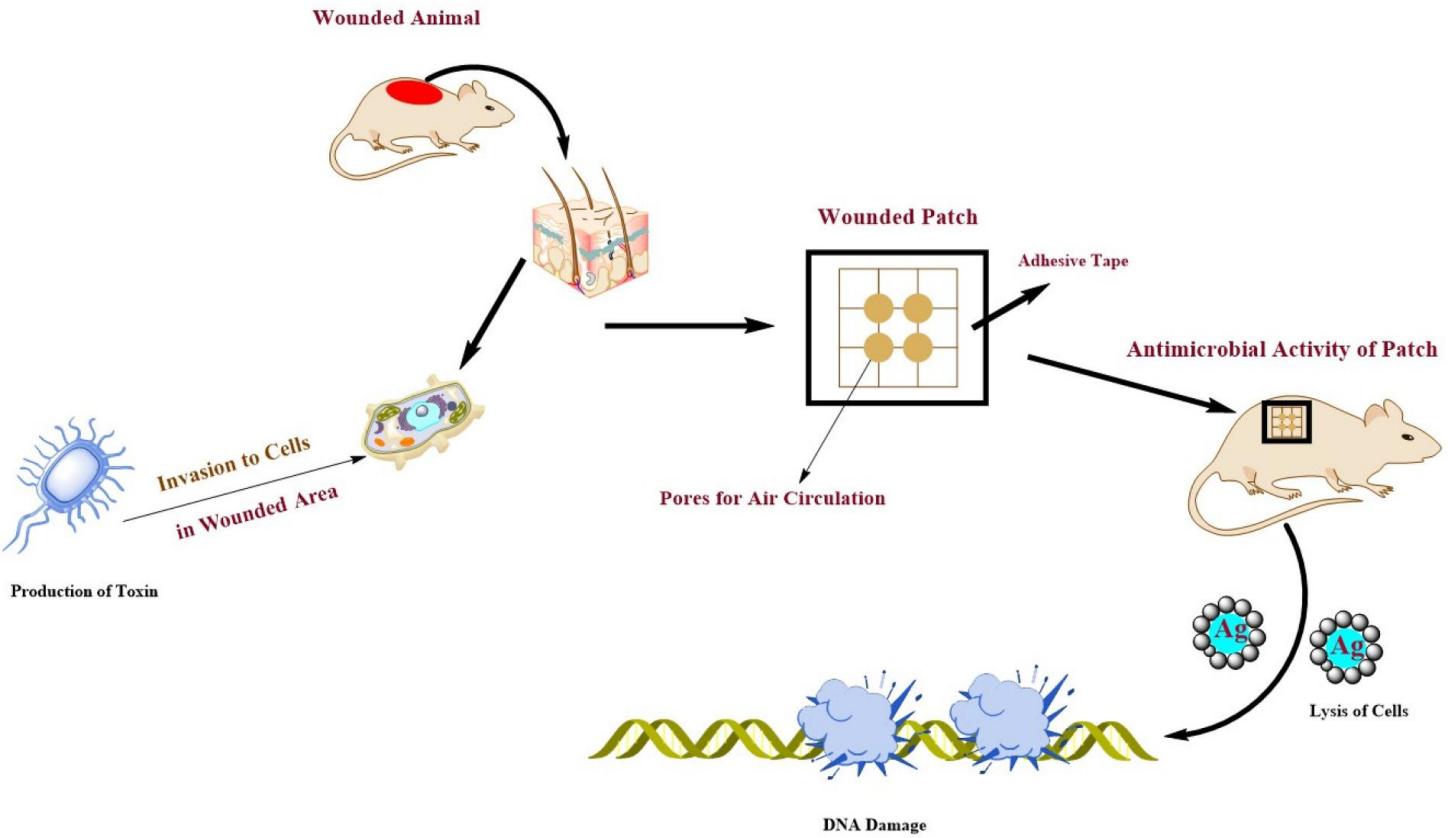
Schematic medication of wounded area by patch including Ag nanoparticles [223]

Anti‐coat was the initial nano‐silver product to be recommended by the pharmaceutical industry to medicate chronic dermal wounds and infections such as pemphigus, ulcers, gangrenes, and other toxic epidermal necrolysis [189]. It was formulated by the Canadian scientist Dr. R. Burrell, and later clinically tested by Chinese scientists. The appraisal demonstrated that topical application of biogenic AgNPs dressings promotes quick re‐epithelialisation along with antimicrobial properties [190,191]. Further studies produced a remedy, which was formulated by the combination of AgNPs and chitosan polymer. This antimicrobial bandaid with low tissue absorbance was used to heal deep wounds [192]. AgNPs have shown a cytocompatibility with tested cell lines. These particles have higher moisture contents and good transparency level, which they are considered as a good option for treating chronic wounds that have higher microbial bioburden [193]. Figure 4 portrays the mechanism of wound healing using patch containing Ag nanoparticles.

### 7.5 Medical implants

A preliminary implantation device that contained elemental silver (Ag^+^) was a prosthetic silicon heart valve, which possessed excellent antimicrobial and anti‐inflammatory potencies [194]. However, the metallic silver coating caused hypersensitivity, thus inhibiting normal function of fibroblasts leading to paravalvular leakage in convalescents [195]. To resolve this drawback, Andara and coworkers, engineered heart valves and stents coated with green AgNPs and diamond‐like carbon. These coated cardiovascular implants proved to be safe and have the desired antimicrobial and anti‐thrombogenic properties [196]. AgNPs precisely provide the sustained exposure to infarcted heart through direct intramyocardial injection [197]. Furthermore, their hemodynamic and mechanical mechanisms were investigated by creating multiple coats of nano‐silver implants [198].

Aside from cardiovascular implants, AgNP‐coated catheters were also extensively investigated to prevent serious catheter‐associated infections. Along this line, there are two commercially available AgNP‐coated catheters that is silver‐line (Spiegelberg GmbH and Co. KG, Hamburg, Germany) and ON‐Q Silver Soaker™ (I‐Flow Corporation, CA, USA). These catheters were accepted worldwide as they were validated to reduce post‐surgical infections while having least or no side effects. In vitro studies on animal models demonstrated that plastic catheters filmed with AgNPs possess bacteriostatic effects up to 72 h [198]. Similarly, clinical studies on 19 nano‐silver‐catheterised convalescents validated clear cerebrospinal fluid cultures along with negative catheter‐associated ventriculitis [199].

### 7.6 Dental implants

**FIGURE 5:**
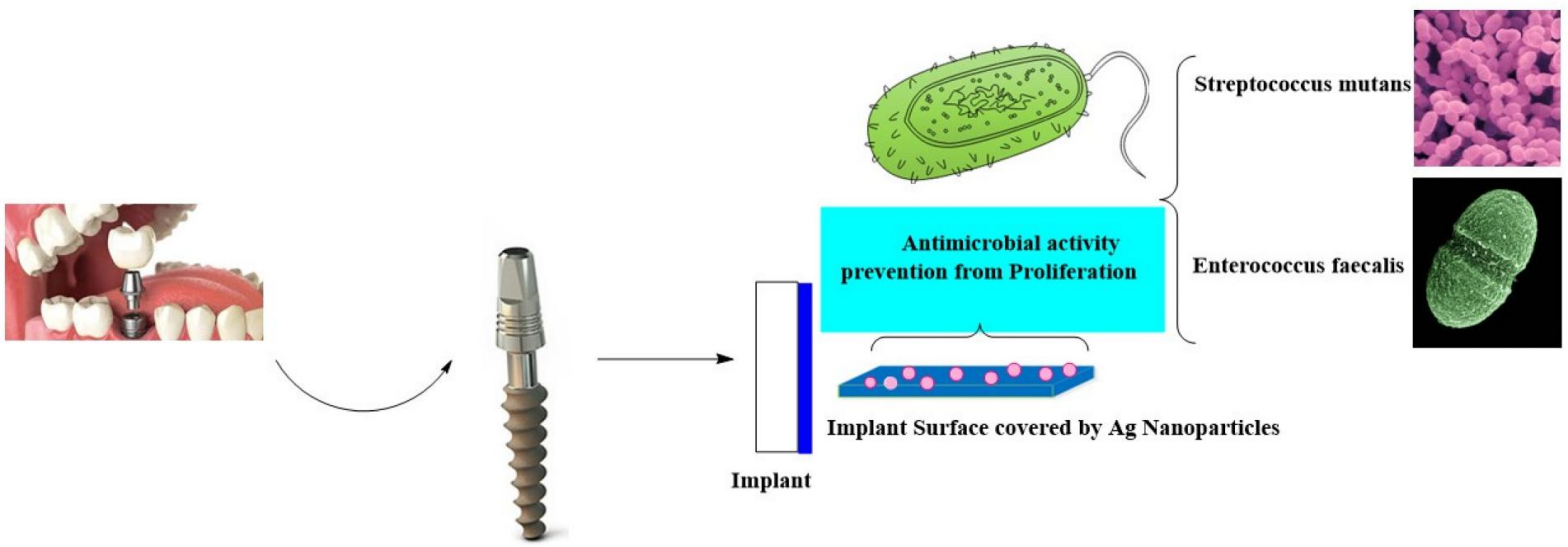
Schematic presentation of an implant surface integrated by Ag nanoparticles and its antimicrobial activity

Biogenic phyto‐AgNPs can be used in dental medicine as part of dental fillers or complex orthodontic implants [200,201]. Incorporation of AgNPs at different concentrations with composite resins proved useful, and the mechanical and antimicrobial properties of the product improved significantly. The endodontic filling material containing AgNPs provide better antibacterial properties against *Enterococcus faecalis*, *Streptococcus milleri*, and *S. aureus*. It inhibits the proliferation of caries‐associated bacteria such as *Streptococcus mutans*, *Streptococcus milleri*, *Enterococcus faecalis*, and in some cases *Staphylococcus aureus* (Figure 5) [202]. A recent study has shown that coating of the silver nanoparticles with silica form Ag@SiO_2_ further improves their antimicrobial activity and no cytotoxicity on human dental cells was observed [203].

### 7.7 Other medical advancements

Biosynthesised AgNPs, as part of biosensors, have recently been used in the diagnosis of cancers and Alzheimer's disease [204]. They are also used as bio‐tags for quantitative analysis of a process, and as carriers of therapeutic agents [205]. Their antimicrobial properties are also used in eye care products such as contact lens solution, and are expected to be used in the next generation of implantable medical devices [206]. *N. khasiana* extract has recently been used for production of silver nanoparticles. Performance analysis of these particles has shown that they can be used for the treatment of Alzheimer's disease [207].

#### 7.7.1 Fabrics

AgNPs have been widely used in diverse consumer products including clothes, laboratory gowns, and socks as well as medical products including dressing bandages, surgical gowns, and facemasks due to their antimicrobial potency. The NPs coating on cloth surface is achieved using numerous techniques including pad dry cure method, sol–gel method, and sonochemical method [208,209]. Several studies have claimed that the NP‐coated fabrics have the potential to protect from ultraviolet radiations as well as their antimicrobial ability [210,211]. Previous studies had shown that coated NPs are particularly suitable in protecting against *S.aureus* and *E.coli* pathogens [212,213].

## 8 AGRICULTURAL APPLICATIONS

**FIGURE 6:**
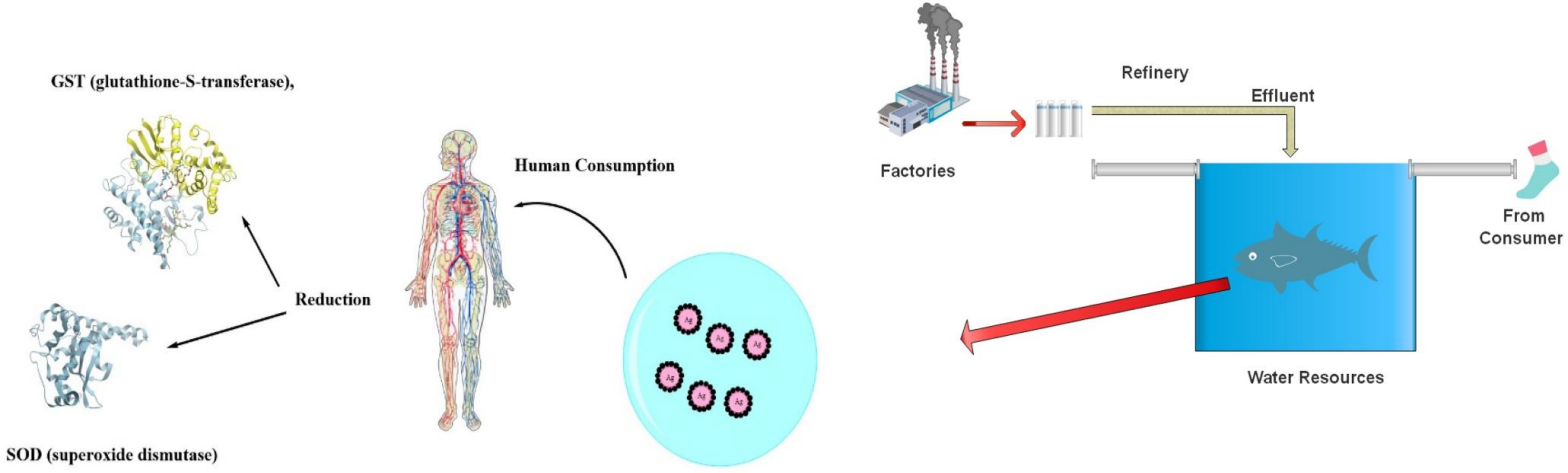
Schematic presentation of accumulation of Ag nanoparticles in marine sources and some of their impacts on humans [224]

AgNPs show promising outcomes in agricultural practices by promoting crop conservation, animal vitality, and fisheries [214]. They function as active biosensors to detect pests/pathogens, monitor packaged products, and standardise the release of reactive substances [215,216]. Biogenic AgNPs capsules are used as positive genetic carriers to transport agricultural ingredients to produce novel stress‐ and disease‐resistant crops plus sensing favourable field environment for excessive crop production (Figure 6) [217,218]. These particles are also being used as antimicrobial as well as ethylene inhibitors in agriculture industry to increase their post‐harvest quality [219]. Recently, *A. aspera* and *S. dulcis* have been used to prepare fungicides [220]. These fungicides can be used as an alternative to synthetic fungicides for agricultural applications.

## 9 ENVIRONMENTAL APPLICATIONS

Published research pertaining to silver nanotechnology revealed that approximately one‐third of environmental silver is dispersed through excessive utilisation of nanoparticles. This silver gets into the atmosphere and wastewater via washing off consumer goods that are loaded with nano‐silver such as cosmetics, toiletries, fabrics, and various household appliances [221]. In addition, silver nanoparticles can be used as catalyst in power generation equipment of automobiles, which reacts with hazardous gases such as nitrous oxide and carbon monoxide to reduce their environmental impacts. Silver nanoparticles can also be used in wastewater treatment plants to remove different types of hazardous pollutants and pathogens from water. They are effective in the removal of microorganisms, minerals and pesticides from wastewater to purify it for drinking purposes [161]. A report on sewage sludge showed that silver has entered water systems and was even found to be aggregated into sediments and soil; however, environmental risks are not well‐characterised. It is presumed that the accumulation of these silver particles in the bodies of aquatic such as mollusks can disturb the food chain and ecosystem. This bioaccumulation can inhibit the growth of beneficial bacteria too. Therefore, new protocols should be developed for qualitative and quantitative analysis of environmentally released silver to protect the biosphere [222]. *Aerva lanata* synthesised AgNPs have been proven effective in degradation of dyes, and have shown potential for environmental applications.

**FIGURE 7:**
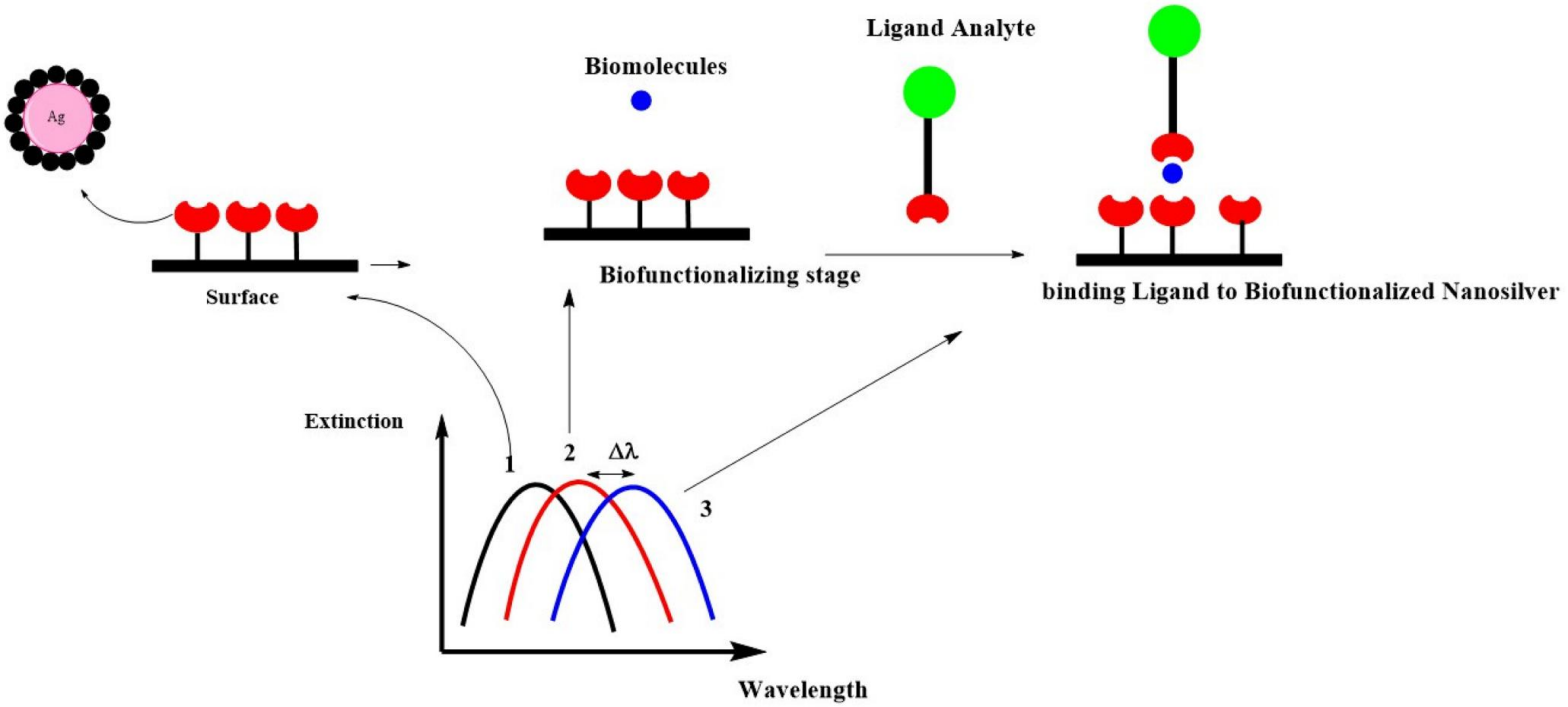
Schematic representation of silver nano‐biosensor: stage 1) stabilisation of silver nanoparticles on the surface, stage 2) Bio‐functionalisation of the surface and an increase in refractive index, stage 3) reaction with a ligand analyte and shifting the blue curve to higher wavelength (showing bio‐sensing activity) [225]

Figure 7 depicts the mechanism of bio‐sensing activity when Ag nanoparticles are deposited on a surface.

## 10 OTHER RECENT ADVANCEMENTS

Green AgNPs can be used to manufacture conductive inks due to their exclusive thermal and electrical conductivities. They are combined with vanadium oxide, which increases shelf‐life and efficiency of batteries. They are also used in optical spectroscopies such as metal‐enhanced fluorescence (MEF) and surface‐enhanced Raman scattering (SERS) due to their increased affinity to capture light energy [225].

## 11 NANO‐SILVER TOXICITY

Due to the increasing prevalence of AgNPs in consumer goods, there is an urgent need to understand their biological mechanisms and hitherto unreported health risks. For centuries, colloidal silver solutions were utilised in folk medicine to treat various chronic infections but the deposition of Ag+ in tissues led to a cosmetic malady called ‘Argyria’ or ‘argyrosis’. Under these conditions, the skin turns blue or bluish‐grey. On the other hand, experimentation on AgNPs led to the finding that nanoparticles are superior over silver salt solutions and colloidal silver because they demonstrated effective antimicrobial properties at less dosage, which can assist in the treatment of infections without any reported side effects.

Research findings showed that AgNPs can act as anti‐inflammatory, genotoxic, mutagenic, cytotoxic, and cytostatic agents. Although these properties might be interpreted as useful for immune potentiation or tumour control for normal cells and DNA, they can still be deleterious. AgNPs can penetrate the cell wall and create oxidative stress, and the resulting reactive oxygen species (ROS) can cause dysregulated enzyme cascades and undesirable immune activation.

## 12 CONCLUSIONS

In summary, findings from this literature review suggest that phyto‐fabrication of AgNPs is an ideal strategy to generate large numbers of eco‐friendly nanoparticles in a cost‐effective way. Based on the AgNPs plasmonic resonance, purified dry pellets of AgNPs have *λ*_max_ values in the range of 350–500 nm, with a variably simple to complex morphologies in the range of 10–500 nm diameters, depending on experimental conditions. These green‐synthesised AgNPs exhibit distinctive biological traits which can be used in assorted pharmaceutical and engineering industries to make commercial novel and effective consumer items. However, more detailed studies related to the toxicity of AgNPs are required to establish their safety and efficacy.
